# Multi-candidate immunohistochemical markers to assess radiation response and prognosis in prostate cancer: results from the CHHiP trial of radiotherapy fractionation

**DOI:** 10.1016/j.ebiom.2023.104436

**Published:** 2023-01-26

**Authors:** Anna Wilkins, Barry Gusterson, Holly Tovey, Clare Griffin, Christine Stuttle, Frances Daley, Catherine M. Corbishley, David Dearnaley, Emma Hall, Navita Somaiah

**Affiliations:** a Division of Radiotherapy and Imaging, The Institute of Cancer Research, London, United Kingdom; b Royal Marsden Hospital, Sutton, United Kingdom; c Clinical Trials and Statistics Unit, The Institute of Cancer Research, London, United Kingdom; d Division of Breast Cancer Research, The Institute of Cancer Research, London, United Kingdom

**Keywords:** Fractionation, Immunohistochemistry, Prostate cancer, Radiotherapy, Treatment stratification

## Abstract

**Background:**

Protein markers of cellular proliferation, hypoxia, apoptosis, cell cycle checkpoints, growth factor signalling and inflammation in localised prostate tumours have previously shown prognostic ability. A translational substudy within the CHHiP trial of radiotherapy fractionation evaluated whether these could improve prediction of prognosis and assist treatment stratification following either conventional or hypofractionated radiotherapy.

**Methods:**

Using case:control methodology, patients with biochemical or clinical failure after radiotherapy (BCR) were matched to patients without recurrence according to established prognostic factors (Gleason score, presenting PSA, tumour-stage) and fractionation schedule. Immunohistochemical (IHC) staining of diagnostic biopsy sections was performed and scored for HIF1α, Bcl-2, Ki67, Geminin, p16, p53, p-chk1 and PTEN. Univariable and multivariable conditional logistic regression models, adjusted for matching strata and age, estimated the prognostic value of each IHC biomarker, including interaction terms to determine BCR prediction according to fractionation.

**Findings:**

IHC results were available for up to 336 tumours. PTEN, Geminin, mean Ki67 and max Ki67 were prognostic after adjusting for multiple comparisons and were fitted in a multivariable model (n = 212, 106 matched pairs). Here, PTEN and Geminin showed significant prediction of prognosis. No marker predicted BCR according to fractionation.

**Interpretation:**

Geminin or Ki67, and PTEN, predicted response to radiotherapy independently of established prognostic factors. These results provide essential independent external validation of previous findings and confirm a role for these markers in treatment stratification.

**Funding:**

10.13039/501100000289Cancer Research UK (BIDD) grant (A12518), 10.13039/501100000289Cancer Research UK (C8262/A7253), Department of Health, 10.13039/501100000771Prostate Cancer UK, 10.13039/100008719Movember Foundation, NIHR Biomedical Research Centre at 10.13039/100012139Royal Marsden/ICR.

**Research in context**

**Evidence before this study**

Protein markers of cellular proliferation, hypoxia, apoptosis, cell cycle checkpoints, growth factor signalling and inflammation in localised prostate tumours have previously shown prognostic value following radiotherapy and thus may aid treatment decisions (i.e. intensification with novel agents versus surveillance) for localised prostate cancer. However, a single combinatorial comparative analysis has not been conducted. Gene expression signatures, including Decipher, can also predict recurrence after treatment for localised prostate cancer but are expensive and may not be feasible with limited tumour material.

In addition, radiotherapy with curative intent is typically given as a uniform dosing schedule with a “one size fits all” approach, there is no biology-based individualised radiotherapy fractionation and an important unanswered question is whether shorter intensive schedules with a lower total dose are as effective as longer schedules that reach a higher total dose across biologically diverse prostate tumours.

**Added value of this study**

The CHHiP trial is the largest trial conducted worldwide to randomise patients with localised prostate cancer between conventional (longer) and moderately hypofractionated (shorter) radical radiotherapy schedules. It is therefore a unique opportunity to identify whether tumour biology should be used to select optimal radiotherapy schedules. Our integrative analysis combined a diverse panel of protein markers known to predict recurrence after prostate cancer radiotherapy and identified that markers of proliferation (Ki67 or Geminin) as well as PTEN loss predicted recurrence independently of prognostic factors currently used in the clinic. These findings did not differ according to fractionation schedule which provides reassurance that shorter kinder radiotherapy schedules can be used irrespective of proliferative status and other biological hallmarks of prostate cancer.

**Implications of all the available evidence**

Our findings suggest that immunohistochemistry for proliferative markers (Ki67 or Geminin) plus PTEN in combination can assist treatment selection for patients considering radiotherapy for prostate cancer. Immunohistochemistry is widely available and affordable, and is feasible with small quantities of tumour material, which facilitates incorporation into clinical care. The increased recurrence in patients with PTEN loss raise the possibility that AKT inhibition in combination with radiotherapy may improve outcomes. Finally, our findings support the ongoing use of shorter less expensive hypofractionated radiotherapy schedules as a standard of care across prostate cancers.

## Introduction

Over 1.4 million men worldwide were diagnosed with prostate cancer in 2020^1^ and in the developed world most cases are diagnosed as localised tumours. Here, external beam radiotherapy, prostatectomy and brachytherapy are widely used radical treatments with the further option of active surveillance for patients with low to intermediate risk disease wishing to avoid treatment toxicities. Treatment stratification for intermediate risk localised prostate cancer is challenging; recurrence rates vary widely,^2^, ^3^, ^4^ yet a substantial proportion of tumours remain well controlled with active surveillance. There is therefore an unmet need for affordable and widely-available biomarkers to guide treatment decisions, including the potential addition of targeted systemic therapy, based on individual tumour biology.

A number of immunohistochemical (IHC) markers have been evaluated in diagnostic biopsies from men recruited to randomised trials of radical radiotherapy to the prostate and shown an association with recurrence. These include Ki67, a well-established marker of cell proliferation,^5^, ^6^, ^7^, ^8^, ^9^, ^10^, ^11^ as well as cell cycle checkpoint proteins p53, Murine Double Minute 2 (MDM2) and p16.^9^^,^^11^, ^12^, ^13^, ^14^, ^15^, ^16^, ^17^, ^18^ P53 is an important tumour suppressor gene with a key role in enabling cells to enter the G1/S checkpoint following DNA damage; p53 is degraded by MDM2. P16 can also trigger G1 cell cycle arrest and functions in the induction of senescence. Hypoxia-inducible factor 1 alpha (HIF1α), vascular endothelial growth factor (VEGF) and osteopontin (OPN) have also been associated with recurrence post-radiotherapy.^19^, ^20^, ^21^ HIF1α has a central role in the cellular response to hypoxia whilst VEGF acts to stimulate angiogenesis. Osteopontin is a bone sialoprotein with diverse functions in tumour progression including signalling via HIF1α and VEGF. Differential expression of the anti-apoptotic protein B cell lymphoma 2 (Bcl-2) and the pro-apoptotic protein Bax have additionally been associated with recurrence following prostate radiotherapy.^10^^,^^12^^,^^13^ Phosphate and tensin homolog (PTEN) is a commonly-mutated tumour suppressor gene which negatively regulates the proto-oncogenic PI3K–AKT–mTOR signalling pathway. Both PTEN loss and epidermal growth factor receptor (EGFR) amplification have shown significant prediction of radiorecurrence in prostate cancer,^19^^,^^22^ as has cyclooxygenase 2 (COX2),^11^ which has a pro-inflammatory function.

IHC is widely available, the technique enables preservation of cellular architecture, and is feasible with very few tumour glands. Despite these advantages, IHC markers have not been incorporated into clinical treatment stratification. This is partly due to a lack of external validation combining multiple markers into a single analysis. Historical limitations in digital pathology have also restricted innovative computational histological analysis in prostate cancer, but progress in artificial intelligence means novel automated image analysis approaches are becoming feasible.^23^, ^24^, ^25^

The CHHiP trial (CRUK/06/016; ISRCTN97182923) randomly assigned 3216 men with localised prostate cancer to conventional fractionation (74Gy in 37 fractions over 7.4 weeks) or one of two hypofractionated schedules (60Gy in 20 fractions over 4 weeks or 57Gy in 19 fractions over 3.8 weeks).^2^ The trial recruited patients between 18th October 2002 and 17th June 2011 with detailed eligibility criteria previously published.^2^ In Trans-CHHiP (CRUKA12518), the main translational substudy within CHHiP, tumour tissue from 2047 UK patients from 107 pathology departments has been collected.^26^ Trans-CHHiP aimed to evaluate the above IHC markers in combination to derive a panel of histological markers that improved treatment stratification. As CHHiP was the largest completed trial to randomise men between standard fractionation and hypofractionation, a second aim was to evaluate if any biomarkers predicted recurrence according to fractionation schedule - this could potentially enable biology-based individualised radiotherapy fractionation, rather than the current “one size fits all” approach.

## Methods

### Study design

In view of the low rate of biochemical or clinical recurrence (BCR) in CHHiP,^2^ a matched case:control methodology was used to select study participants (the detailed eligibility criteria for CHHiP trial entry is outlined in the main trial report^2^). Patients with BCR (cases) were matched 1:1 to patients without BCR (controls) using the matching criteria of fractionation schedule and established prognostic factors including PSA (<10/10–20/>20 ng/mL), centrally-assigned Gleason grade group (3 + 3/3 + 4/4 + 3/4 + 4), and T stage (T1/T2/T3).

A specialist uropathologist (CMC) centrally reviewed all tissue samples, including assignment of Gleason grade group and classification using International Society of Urological Pathology (ISUP) guidelines.^27^

### Ethical approval

All patients providing samples to Trans-CHHiP provided written informed consent. The study, including the Trans-CHHiP consent form was approved by the London Multi-centre Research Ethics Committee (04/MRE02/10). The study was designed in accordance with the TRIPOD guidelines (see supplementary TRIPOD checklist).

### Immunohistochemistry staining and scoring

IHC staining and scoring was performed in two parts. For IHC part one, all fifteen markers were stained and scored in a cohort of 110 tumours balanced for BCR (n = 55) versus no BCR (n = 55) (Tables 1 and Table S1). Each marker was evaluated for staining quality and variation in signal across tumours with the aim of excluding any markers failing to show robust staining in prostate biopsies, or those with a uniform non-discriminatory signal across tumours. For IHC part two, a refined list of markers with reproducible staining and variation in signal between samples was evaluated in the remaining 325 tumours.

**Table 1:** IHC biomarkers including clone and specific scoring methodology used in Trans-CHHiP.

Biomarker	Antibody clone, dilution	Scoring methodology
**Cell proliferation**		
Ki67 mean	Monoclonal (MIB1), 1:300	Global unweighted method based on 4 high power fields that represent intratumoural Ki67 heterogeneity (final score was average of two scoring investigators)^25^
Ki67 max		Maximum score of 4 high power fields scored as per Ki67 mean^25^
Geminin	Polyclonal, 1:2000	Percentage positive cells per high power field in up to 10 fields^28^
**Cell cycle checkpoints**		
P53	Monoclonal (DO7), 1:200	Intensity of staining (0, 1, 2 or 3) and proportion of cells staining positive (0, <1, 1–60, >60).^29^ Mutant = >60% strong staining. Clonal mutant designated if > 100 cells strong staining but less than 50% of all tumour cells (see supp Fig. S1)
MDM2	Monoclonal (IF2), 1:400	Nuclear intensity (0, 1, 2 and 3) of tumour versus normal prostate
P16	Monoclonal (E6H4), Ventana system	<25% versus ≥25% nuclei positive
P-chk1	polyclonal, 1:300	Average number of nuclear foci per tumour cell
**DNA damage response**		
ATM	Monoclonal (Y170), 1:150	Nuclear intensity (0, 1, 2 and 3) of tumour versus normal prostate^30^
MRE11	Monoclonal (12D7), 1:3000	Nuclear intensity (0, 1, 2 and 3) of tumour versus normal prostate^30^
**Growth factor signalling**		
PTEN	Monoclonal (6H2.1), 1:100	H-score ≤10 defined PTEN loss.^31^ H-score was a multiplication of percentage positivity (range 0–100%) and intensity of staining (0 nil, 1 weak, 2 moderate, 3 strong). Scored mutant if >50% cells had H-score <10. Scored clonal mutant if > 100 cells showed H-score ≤10 in <50% of tumour cells (see Fig. 1).
EGFR	Monoclonal (E7), 1:60	Membranous staining intensity (0, 1, 2 and 3) of tumour versus normal prostate (DAKO EGFR scoring manual)
**Apoptosis**		
Bcl-2	Monoclonal (124), 1:100	Any tumour cell cytoplasmic staining was designated increased expression^12^
**Hypoxia**		
HIF1α	Monoclonal (clone 54), 1:200	H-score based on nuclear intensity (0, 1, 2 and 3) and nuclear percentage staining positivity^19^
VEGF	Polyclonal, 1:250	Cytoplasmic staining intensity (0, 1, 2 and 3) of tumour versus normal prostate^20^
OPN	Polyclonal, 1:2000	Cytoplasmic staining intensity (0, 1, 2 and 3) of tumour versus normal prostate^20^
**Inflammation**		
COX2	Monoclonal (CX-294), 1:200	Cytoplasmic staining intensity (0, 1, 2 and 3) of tumour versus normal prostate^11^

IHC staining was performed using full-face diagnostic sections (see Table S1 for detailed staining protocols). In brief, after heat-mediated antigen retrieval, slides were placed on a DAKO link Autostainer and stained using a pre-programmed standard two step method. This consisted of 1 hour incubation with the relevant antibody, followed by Dako Flex Envision HRP and DAB chromagen. Slides were counterstained in Gills Haematoxylin prior to dehydration, clearing and coverslipping. An adjacent section from each biopsy block was stained with the basal marker CK5/6 (DAKO (D5/16), dilution 1:75, antigen retrieval DAKO PT module pH9.0) to help distinguish pre-invasive from invasive disease.

**Fig. 1:**
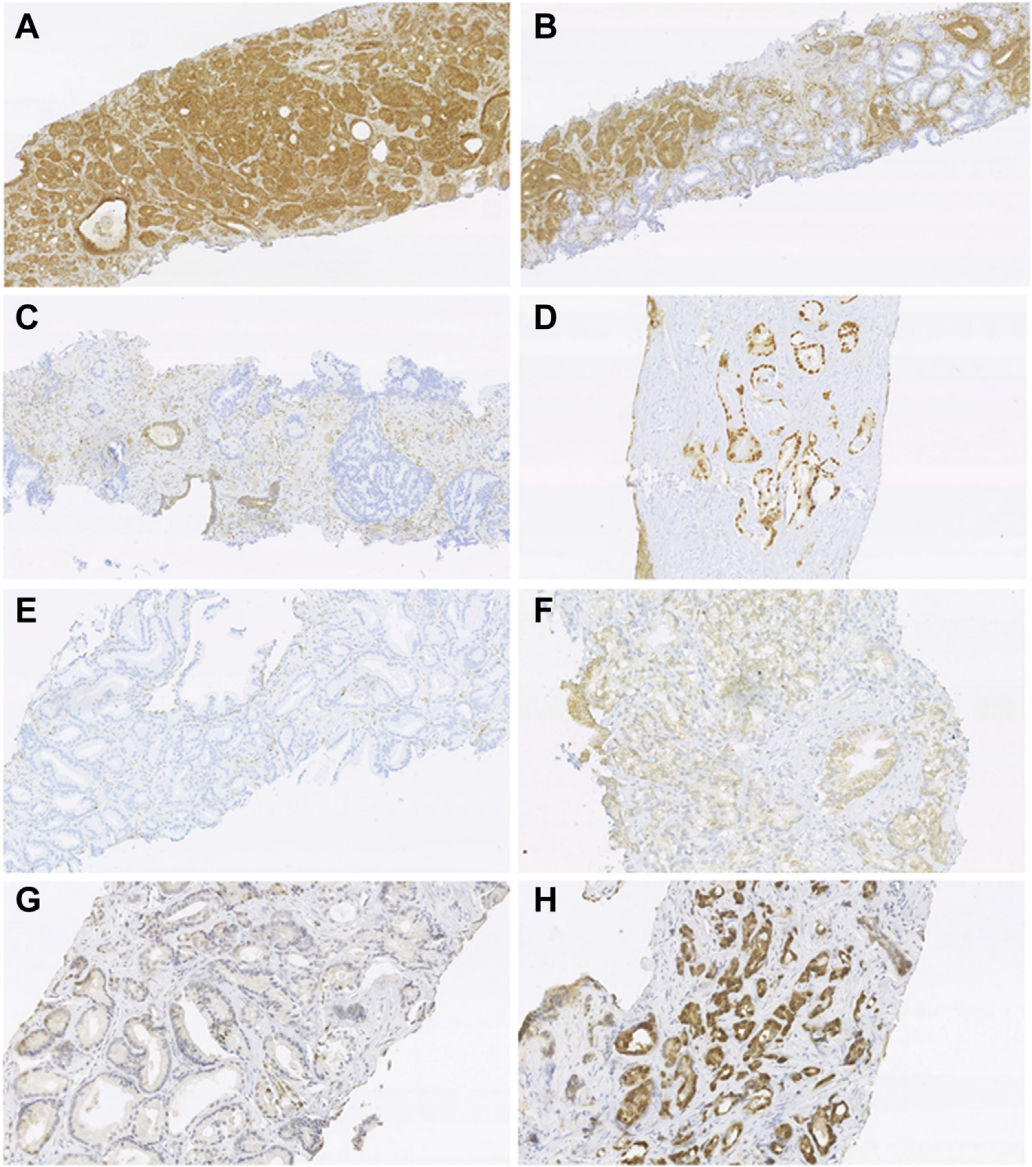
**Staining patterns of IHC markers scored as categorical variables including PTEN, p16, p53 and Bcl-2.** A: wild-type PTEN tumour, B: focal (clonal) loss of PTEN in tumour surrounding by wild-type tumour glands, C: PTEN loss throughout tumour areas without loss in non-malignant glands, D: p53 mis-sense mutant. E: No cytoplasmic expression of Bcl-2. F: increased cytoplasmic expression of Bcl-2 in tumour. G: low p16 nuclear staining in tumour (<25%). H: high p16 nuclear staining (>75%). A–H:20X.

Slides were scored using bright field microscopy by two independent investigators who were blinded to BCR status and radiotherapy fractionation schedule. The specific scoring methodology was derived from the relevant published literature^12^^,^^19^^,^^28^, ^29^, ^30^, ^31^, ^32^, ^33^ (Table 1) and typically used the normal prostate stroma and glands as a reference in each section evaluated. A minimum of 100 tumour cells was required to attribute a score per tumour. Prostatic intra-epithelial neoplasia (PIN) and intraductal carcinoma (IDC) were not scored. Some markers including PTEN, p53 and p16 showed distinct spatial differences in expression within a single tumour, indicating a tumour clone with differing biomarker status to the remaining tumour. Therefore, a category of “focal loss/gain” was defined as loss/gain in more than 100 tumour cells but less than 50% of all the tumour area, in contrast to “total loss/gain” where more than 50% of tumours cell had loss/gain of the relevant biomarker. This is demonstrated for PTEN in Fig. 1 and for p53 in Fig. S1. A formal statistical review of agreement in scores was undertaken after IHC part one. For continuous variables of Ki67 and Geminin, any cases with a discrepancy >10% were re-scored to ensure scores were within 10% of each other. For all other markers, both investigators reviewed and reconciled discrepant scores.

### Study endpoints

Scores for the individual IHC markers were evaluated as separate study endpoints. All recurrence data was derived from a CHHiP data snapshot dated October 2020 where median follow was 9.2 years (interquartile range 8.2–11.0 years). Recurrence (BCR) was defined as biochemical failure using the Phoenix definition^34^ and/or clinical failure after radiation therapy. Non-recurrence was defined in patients with no evidence of BCR who were alive at the time of data snapshot. For the prognostic evaluation, all patients experiencing BCR were considered. For evaluation of prediction of recurrence according to fractionation schedule, patients experiencing BCR within two years of radiation start were excluded because they were more likely to have distant metastases at the time of radiotherapy than local in-field recurrence.

### Statistical analysis

Agreement in IHC scores in part 1 between the two investigators was measured either using the concordance correlation coefficient (Ki67) or Cohen's kappa statistic (all other scoreable biomarkers except p-chk1 which was scored by one investigator). Imputation of missing biomarker values to enable sensitivity analyses was not carried out because the different biomarkers address diverse aspects of tumour biology. This meant it was difficult to reliably use findings from one biomarker as the basis for imputation of other missing biomarker values.

Using the entire Trans-CHHiP case–control study sample with complete laboratory data for each biomarker separately, conditional logistic regression models were fitted to estimate the prognostic value of the biomarkers on the risk of BCR. Age at randomisation was included in each model which was also adjusted for matching strata. The Benjamini-Hochberg procedure with a false discovery rate (FDR) of 10% adjusted for multiple testing was applied. Subsequently, biomarkers which were significant prognostic markers in univariable analysis were included in a multivariable model. This multivariable model was based on patients with complete biomarker data. 1:1 matching was maintained by random selection of recurrences from within the strata to match 1:1 with the controls, where there were more recurrences than controls within a stratum. Of note, no imputation of missing data was carried out.

To determine whether any biomarker predicted an impact of fractionation schedule on BCR, a biomarker:fractionation interaction term was included in the multivariable models. An indicative power calculation has been previously described and estimated the study would have a power of 70%–75.5% (with an alpha of 0.017) to detect an interaction between fractionation schedule and Ki67.^35^ All statistical analysis was conducted using STATA version 13.0 (StataCorp, College Station, TX).

### Role of the funders

The funding sources had no role in the conduct or reporting of the research.

## Results

### Patient characteristics

435 patients were included in the initial matched case:control cohort. Of these, valid biomarker scores were derived for a maximum of 336 matched patients (range over the 8 biomarkers: 276–336). Up to 159 patients (range 99–159) were excluded due to either no suitable case or control for matching, or insufficient tumour for sectioning (e.g. near block exhaustion) or scoring (<100 tumour cells). Patients selected for the case–control study for whom biomarkers could not be evaluated were slightly more likely to be lower risk category and lower Gleason grade compared to patients for whom biomarkers were evaluable. This is likely due to these being smaller tumours with less tissue available for analysis, however, cases and controls were matched by risk factors (including Gleason grade) and this was adjusted for in analyses.

Tables S2–S9 show the distribution of matching variables according to fractionation schedule for the eight biomarkers scored in part two of Trans-CHHiP. For the multivariable analysis, 293 patients had complete biomarker data for the three biomarkers included in the multivariable model (152 cases with recurrence and 141 controls). Some controls had no matching recurrence case, and there were more recurrences than controls for some strata. Therefore, to maintain 1:1 matching, a cohort of 212 patients were evaluated in the multivariable analysis including 106 cases with BCR and 106 controls without BCR. Table 2 shows the distribution of matching variables according to fractionation schedule in patients with available scores for the biomarkers included in the multivariable model, and in the entire CHHiP cohort.

**Table 2:** Distribution of matching variables according to fractionation schedules for multivariable dataset, note matching variables for individual biomarkers are shown in Tables S2–S9 (PSA: Prostate specific antigen).

Multivariable analysis dataset								
	**74Gy/37f**		**60Gy/20f**		**57Gy/19f**		**Total**	

	**N = 84**		**N = 62**		**N = 66**		**Total N = 212**	

	**N**	**%**	**N**	**%**	**N**	**%**	**N**	**%**

**PSA (ng/ml)**								
<10	24	28.6	28	45.2	24	36.4	76	35.8
10−<20	58	69	30	48.4	36	54.5	124	58.5
≥20	2	2.4	4	6.5	6	9.1	12	5.7
**Tumour stage**								
T1	14	16.7	14	22.6	22	33.3	50	23.6
T2	62	73.8	46	74.2	40	60.6	148	69.8
T3	8	9.5	2	3.2	4	6.1	14	6.6
**Gleason grade**								
3 + 3	6	7.1	4	6.5	12	18.2	22	10.4
3 + 4	44	52.4	40	64.5	40	60.6	124	58.5
4 + 3	26	31	12	19.4	10	15.2	48	22.6
8+	8	9.5	6	9.7	4	6.1	18	8.5
Total	84	100	62	100	66	100	212	100
**Entire bio-banked CHHiP cohort**								
	**74Gy/37f**		**60Gy/20f**		**57Gy/19f**		**Total**	

	**N = 1065**		**N = 1074**		**N = 1077**		**Total N = 3216**	

	**N**	**%**	**N**	**%**	**N**	**%**	**N**	**%**

**PSA (ng/ml)**								
<10	511	48	518	48.2	536	49.8	1565.00	48.7
10-<20	488	45.8	483	45	474	44	1445.00	44.9
≥20	66	6.2	73	6.8	65	6	204	6.3
Missing	0	0	0	0	2	0.2	2	0.1
**Tumour stage**								
T1	356	33.4	422	39.3	393	36.5	1171.00	36.4
T2	623	58.5	561	52.2	582	54	1766.00	54.9
T3	85	8	90	8.4	101	9.4	276	8.6
Missing	1	0.1	1	0.1	1	0.1	3	0.1
**Gleason grade**								
3 + 3	121	11.4	136	12.7	149	13.8	406	12.6
3 + 4	339	31.8	344	32.0	333	30.9	1016.00	31.6
4 + 3	114	10.7	89	8.3	95	8.8	298	9.3
8+	44	4.1	32	3.0	37	3.4	113	3.5
No rescore^a^	447	42.0	473	44.0	463	43.0	1383.00	43.0

a Central rescore for Gleason grade group was carried out by a specialist uropathologist for 1833 tumours.

### Scoring agreement and selection of markers for CHHiP IHC part two

The agreement in IHC scores for markers that could be scored reproducibly between the two independent investigators for part 1 of the study is shown in Tables S10 and S11. Cohen's kappa for agreement ranged from 0.86 (SE 0.06) for Geminin to 1.00 (SE 0.08) for HIF1α. The concordance correlation coefficient for Ki67 has been previously reported.^35^

Seven markers were excluded following part one of Trans-CHHiP IHC: ATM, MRE11 and VEGF showed minimal differences in staining between normal prostate and tumour across all cases (Fig. S2); MDM2 showed non-reproducible staining that was difficult to score consistently; for EGFR, upregulation was only seen in a small minority of cases (4 of 110 (3.6%); Finally, COX2 and OPN showed highly heterogeneous staining patterns across normal prostate and tumour hence were difficult to score consistently (Fig. S2).

**Fig. 2:**
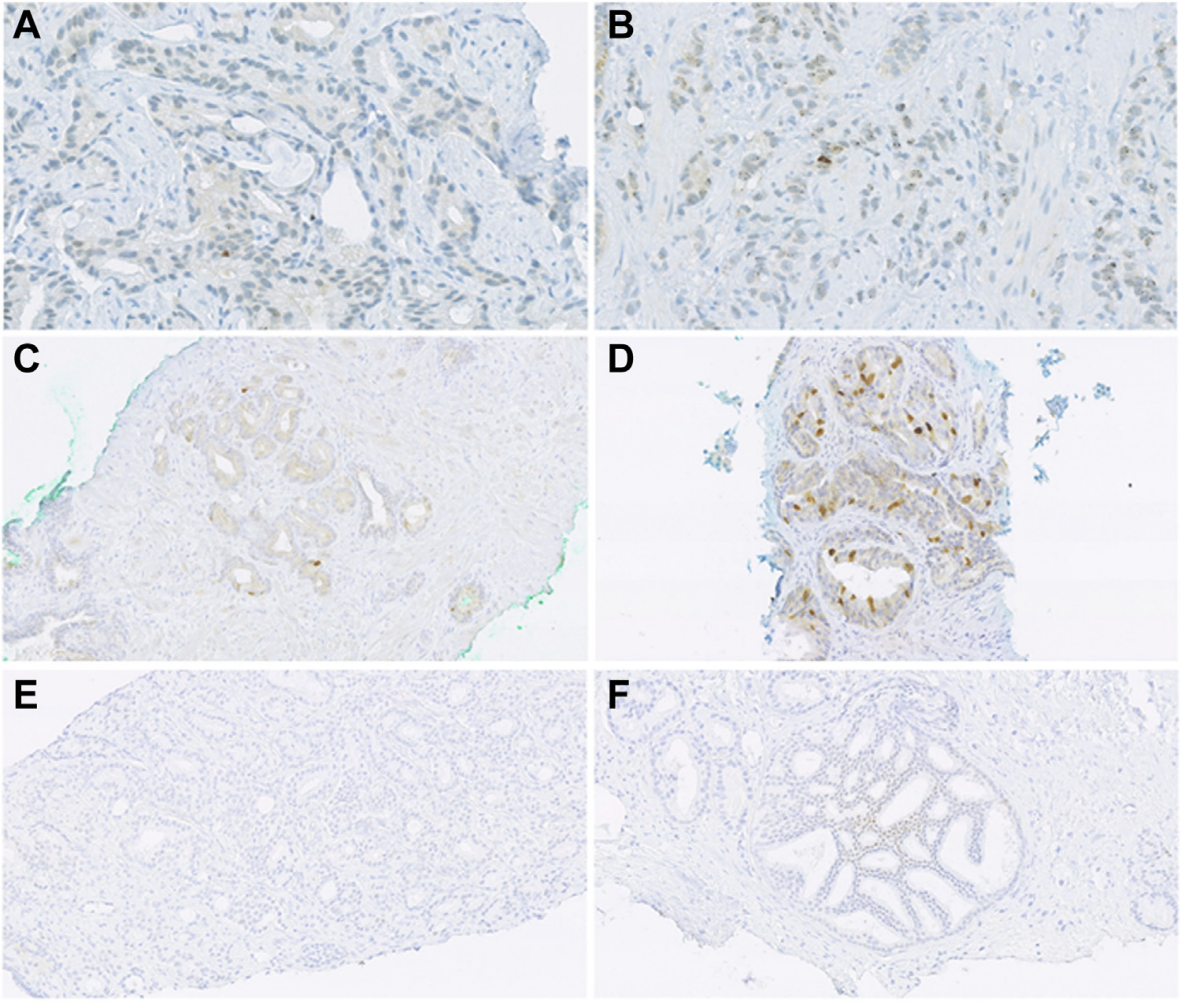
**Staining patterns of IHC markers scored as continuous variables including HIF1α, Geminin and p-chk1 (note Ki67 images for Trans-CHHiP have been previously published).**^35^ A: Minimal p-chk1 staining in tumour. B: High number of p-chk1 foci in tumour cells, C: Tumour with few Geminin positive nuclei, D: Tumour with many Geminin positive nuclei, E: minimal HIF1α nuclear staining in invasive tumour F: HIF1α nuclear staining in intra-ductal carcinoma. A–B: 40X, C–F: 20X.

Fig. 1, Fig. 2 show examples of staining patterns of the eight markers selected for IHC part two. Fig. 1 shows markers scored as categorical variables including PTEN, p16, p53 and Bcl-2. The increased p53 staining shown typically occurs following missense mutation, loss of staining due to any potential nonsense mutations was not scored. Fig. 2 shows markers scored as continuous variables including HIF1α, Geminin and p-chk1 (Ki67 staining has been previously demonstrated^35^). Very few cases showed positive HIF1α nuclear staining; which was typically seen in IDC or PIN (Fig. 2F).

The distribution of IHC scores between cases and controls, and according to fractionation schedule, for PTEN, mean Ki67 and Geminin are shown in Table 3A and B. Tables S12 and S13 show the distribution of IHC scores between cases and controls for the remaining markers (Bcl-2, p16, p53, HIF1α and p-chk1).

**Table 3:** Distribution of IHC scores between cases (with biochemical or clinical recurrence) and controls (without biochemical or clinical recurrence) and according to fractionation schedule for markers included in the multivariable model including PTEN (3A), mean Ki67 and Geminin (3B) .

3A																
PTEN	74Gy/37f (n = 96)				60Gy/20f (n = 92)				57Gy/19f (n = 112)				Total (n = 300)			
	Controls		Cases		Controls		Cases		Controls		Cases		Controls		Cases	
	(n = 48)		(n = 48)		(n = 46)		(n = 46)		(n = 56)		(n = 56)		(n = 150)		(n = 150)	
	N	%	N	%	N	%	N	%	N	%	N	%	N	%	N	%
Wild type	32	66.7	20	41.7	34	73.9	25	54.4	39	69.6	25	44.6	105	63.6	70	46.6
Focal loss	9	18.8	7	14.6	2	4.4	5	10.9	9	16.1	12	21.4	20	15.36	24	19.5
Total loss	7	14.6	21	43.8	10	21.7	16	34.8	8	14.3	19	33.9	25	21.2	56	33.9
	0.007				0.132				0.019				<0.001			

3B								
Biomarkers	Fractionation schedules	Events	N	Median score	IQR (score)		Mean score	SD (Score)
					Q1	Q3		
Mean Ki67	74Gy/37f	Controls	58	6	4	9	6.71	3.42
	(n = 116)	Cases	58	6.38	3.6	9.5	7.61	4.89
	60Gy/20f	Controls	49	6.63	3.38	8.75	6.75	3.72
	(n = 98)	Cases	49	8.13	3.38	11.14	8.29	5.15
	57Gy/19f	Controls	61	5.13	3.33	9.75	6.42	4.18
	(n = 102)	Cases	61	6.75	4.63	10.63	7.89	4.59
Geminin	74Gy/37f	Controls	52	2.65	1.63	4.82	3.48	2.72
	(n = 104)	Cases	52	3.66	2.2	6	4.6	3.46
	60Gy/20f	Controls	47	3	1.5	6	4.27	3.85
	(n = 94)	Cases	47	5.3	2	7.8	5.73	4.87
	57Gy/19f	Controls	55	3	1.2	5.2	4.23	5.24
	(n = 110)	Cases	55	3.33	2	6.95	5.28	5.65

### Prognostic indicators of BCR

PTEN, Geminin, mean Ki67 and max Ki67 were prognostic for BCR after adjusting for multiple comparisons (10% FDR). These markers were fitted in a multivariable model where PTEN and Geminin retained prognostic significance. Table 4 shows the odds ratios for BCR estimated by univariable and multivariable conditional logistic regression models for all biomarkers. Of note, the odds ratio of 2.70 (95% CI 1.66–4.40) for PTEN loss versus PTEN wild type indicates that tumours with PTEN loss had almost three times the odds of recurrence of patients with PTEN wild type tumours. Similarly, the odds ratio of 1.08 (95% CI 1.02–1.14) for Ki67 indicates that a 1% increase in Ki67 staining resulted in an 8% relative increase in the odds of recurrence. Similarly, the odds ratio of 1.07 (95% CI 1.01–1.13) for Geminin indicates that a 1% increase in Geminin staining resulted in a 7% relative increase in the odds of recurrence. Furthermore, a 10% increase in Ki67 or Geminin resulted in an 80% or 70% increase in the odds of recurrence, respectively. Ki67 staining protocols are well established in routine pathology departments and the IHC is more straightforward than Geminin, which required an additional protein block step. To assess the comparative effects of Ki67 and Geminin in the multivariable model, Geminin was removed from the multivariable model and the model was refitted followed by an evaluation of goodness of fit (Table 4). Here, mean Ki67 showed significant prediction of BCR and PTEN loss showed a trend towards significant prediction of BCR. Comparison of the models with and without Geminin with a likelihood ratio test gave a p-value of 0.026 indicating that Geminin significantly improves the fit of the model.

**Table 4:** Odds ratio for biochemical or clinical recurrence estimated by univariable and multivariable conditional logistic regression models for all biomarkers (OR: odds ratio, BH: Benjamini Hochberg).

UNIVARIABLE ANALYSIS				
Biomarker			OR (95% Confidence Interval)	Wald test
				p-value (BH)
Categorical	P53 (n = 328)	Wild type	1.00	
		Mutant	1.19 (0.40–3.56)	0.75 (0.089)
	P16 (n = 276)	Wild type	1.00	
		Mutant	0.65 (0.40–1.04)	0.07 (0.056)
	PTEN (n = 300)	Wild type	1.00	
		Mutant	2.70 (1.66–4.40)	<0.001 (0.011)
	Bcl 2 (n = 310)	No increase	1.00	
		Increase	1.03 (0.50–2.08)	0.95 (0.10)
Continuous	Geminin (n-308)		1.07 (1.01–1.13)	0.02 (0.044)
	HIF1a (n = 310)		1.03 (0.97–1.10)	0.38 (0.078)
	p-chk1 (n = 298)		0.79 (0.52–1.20)	0.26 (0.067)
	Mean Ki67 (n = 256)		1.08 (1.02–1.14)	0.01 (0.022)
	Max Ki67 (n = 256)		1.05 (1.01–1.09)	0.01 (0.033)
**MULTIVARIABLE ANALYSIS including all IHC biomarkers significant on univariable analysis (n = 212)**				
	PTEN	Wild type	1.00	
		Mutant	1.85 (1.02–3.35)	0.04
	Geminin		1.12 (1.01–1.24)	0.04
	Mean Ki67		1.06 (0.98–1.14)	0.15
**MULTIVARIABLE ANALYSIS with Geminin removed from model (n = 212)**				
	PTEN	Wild type	1.00	
		Mutant	1.70 (0.95–3.03)	0.07
	Mean Ki67		1.10 (1.03–1.18)	0.01

### Prediction of fraction sensitivity

The predictive value of all eight biomarkers, according to fractionation schedule, was tested using a biomarker–fractionation interaction test following conditional univariable logistic regression. No statistically significant relationship was seen between any biomarker and BCR according to fractionation schedule (Tables S14–S15).

## Discussion

This immunohistochemical study assessed markers of cellular proliferation, cell cycle phase and checkpoints, growth factor signalling, DNA repair, apoptosis, hypoxia and inflammation in the diagnostic biopsies of patients receiving radical prostate radiotherapy in the CHHiP trial. We demonstrate that Ki67, Geminin and PTEN predict BCR independently of Gleason, PSA and T-stage as cohorts were matched for these routinely-used prognostic parameters. These finding validate several earlier studies showing that proliferative markers and PTEN loss predict recurrence following radiotherapy.^5^, ^6^, ^7^, ^8^, ^9^, ^10^, ^11^^,^^22^ We additionally show that markers of proliferation and PTEN predict BCR independently of each other and established clinical factors indicating that both can aid treatment stratification in the clinic.

Of further clinical relevance, the lack of significant interaction between these predictive markers and radiotherapy fractionation schedule provides important reassurance that shorter, kinder, moderately hypofractionated radiotherapy schedules can be used safely irrespective of proliferative status and other key biological hallmarks of prostate cancer biology. Currently-available toxicity and efficacy outcomes of randomised studies indicate equivalence between profound and moderate hypofractionation, although the efficacy results of PACE-B are awaited.^36^, ^37^, ^38^, ^39^ Stereotactic body radiotherapy (SBRT) schedules of five to seven fractions is moving towards becoming a standard of care for radical radiotherapy to the prostate. Our findings provide reassurance that the “one size fits all” approach is valid across standard fractionation and moderate hypofractionation. Future planned work will be to explore biomarkers that could potentially enable personalised SBRT vs non-SBRT fractionation using samples and clinical outcomes from the PACE trial.

This study cohort consists predominantly of patients with intermediate risk localised prostate cancer where treatment/surveillance decisions are challenging and there is a need for widely-affordable prognostic tools. Single IHC markers such as PTEN have shown comparable prediction of metastases or death from prostate cancer recurrence to established gene expression signatures.^40^ Immunohistochemistry assays and RNA-based genomic signatures each have distinct advantages and Iimitations in aiding treatment decisions; ongoing comparison is important to maximise their complementary use in the clinic for patient benefit. The prostate tumour content varies widely in diagnostic biopsies but diagnosis is not uncommonly based on a small number of tumour glands where extraction of adequate nucleic acid for genomic profiling is challenging.

The single cell resolution of IHC enables evaluation of intra-tumoural heterogeneity, which is known to be relevant for Ki67 in prostate cancer.^35^ IHC also enables evaluation of subclonal changes, which may occur in a minority of tumour glands and is particularly relevant for PTEN where subclonal loss is not uncommon.^41^ A clinical grade IHC assay for PTEN was able to identify cases with heterogeneous *PTEN* gene deletion in a subset of tumour glands as well as loss of PTEN protein in tumours with normal copy number, which potentially arose due to epigenetic or microRNA-mediated mechanisms.^41^ The IHC assays used in this study are straightforward and inexpensive to perform in routine pathology laboratories. Ki67 staining is widely used across tumour sites, with comprehensively evaluated scoring methodologies - clinical use is therefore likely to be easier than Geminin. Furthermore, recent innovation in digital pathology means automated scoring algorithms using continuous variables for both proliferative markers and PTEN are increasingly feasible.^24^^,^^42^

Important negative findings from this study include that markers of hypoxia and apoptosis did not predict recurrence, in contrast to previous reports, including from the RT01 trial.^12^^,^^20^ An association between hypoxia and genomic instability is well-recognised to lead to an aggressive tumour phenotype, especially in tumours with cribiform changes and/or IDC.^43^ IDC was not scored in this study and the eligibility criteria for CHHiP are likely to have excluded patients with larger more hypoxic tumours. Studies evaluating Bcl-2 in predicting recurrence following radiotherapy in prostate cancer have shown inconsistent results.^44^ The use of tissue microarray in RT01 versus full-face sections in this study may also have contributed to differing findings. Other proteins with anti-apoptotic functions, such as Mcl-1 and Bcl-xl, are known to contribute to therapy resistance in prostate cancer.^45^, ^46^, ^47^, ^48^ Robust evaluation of the apoptosis pathways to predict therapy outcome is likely to require assessment of multiple pro- and anti-apoptotic proteins in combination.^49^

Our findings support treatment intensification with either radiotherapy dose escalation or intensified concomitant systemic therapy for patients showing high proliferation (using Ki67 or Geminin) or PTEN loss in the context of a clinical trial. The fairly common finding of PTEN loss (41.7%), plus the significant association with recurrence, suggests a potential role for AKT inhibition alongside androgen deprivation therapy (ADT) and radiotherapy in patients with prostate tumours showing PTEN loss. AKT inhibition is showing considerable promise in castration-resistant prostate cancer.^50^ Reciprocal feedback regulation of PI3K signalling and androgen receptor signalling has been shown in PTEN deficient castration-sensitive prostate cancer where combined inhibition of AKT and androgen receptor signalling was particularly efficacious.^51^ The well-established synergy between ADT and radiotherapy may be further enhanced by AKT inhibition in selected patients; IHC for PTEN could help screening for such patients.

We acknowledge limitations to this study which include a lack of automation of IHC scoring; this would accelerate translation to the clinic and is ongoing work. Secondly, our PTEN protocol has shown a very high concordance with genomic loss of this gene,^32^ however, ideally we would corroborate our findings with genomic sequencing. A further limitation is that we did not adjust for differences in follow-up time between the groups in our analyses, or match patients based on follow-up times. Patients with a recurrence event are typically censored at the time of this event; the average follow up time in patients without a recurrence event was more than double the time to event time in patients with an event suggesting that inadequate follow up in the non-recurrence group is unlikely to have significantly impacted findings.

In conclusion, we show that IHC for PTEN and the proliferative markers Ki67 and Geminin can predict recurrence following prostate cancer radiotherapy independently of established risk factors such as Gleason grade group, presenting PSA and T-stage. Our prognostic models were most significant with PTEN and Geminin, however, Ki67 may be more practical to use in the clinic than Geminin. Our study provides validation of several historical studies in an integrated analysis which uniquely incorporates diverse aspects of prostate cancer biology using clinical outcomes from a large randomised trial. It also provides reassurance that shorter, kinder, moderately hypofractionated schedules can be used safely irrespective of prostate tumour biology. Clinical use of these IHC markers could substantially help meet the current unmet need for widely available and affordable prognostic tools to assist treatment stratification in localised prostate cancer.

## Contributors

Conceptualisation: AW, BG, DD, EH, NS; Methodology: AW, BG, DD, EH, NS, HT, CG; Investigation & Data Collection: AW, BG, CS, CMC; Statistical analysis: HT, CG, EH; Supervision: FD, NS, DD, EH; Writing Original Draft: AW; Writing Review & Editing, AW, BG, HT, CG, CS, FD, CMC, DD, EH, NS.

## Data sharing statement

Raw data for this study were generated at the ICR Clinical Trials and Statistics Unit and the ICR Experimental Histopathology Department. De-identified participant data supporting the findings of this study are available from the corresponding author following publication and after approval of a signed data access agreement.

## Declaration of interests

DD reports personal fees from The 10.13039/501100000650Institute of Cancer Research, during the conduct of the study; In addition, DD has a patent EP1933709B1 issued.

EH reports grants from 10.13039/501100000289Cancer Research UK, during the conduct of the study; grants from 10.13039/100016226Accuray Inc., grants from 10.13039/100007210Varian Medical Systems, grants and non-financial support from Astra Zeneca, grants and non-financial support from 10.13039/100015756Janssen-Cilag, grants and non-financial support from 10.13039/100004326Bayer, grants from 10.13039/100015277Roche Products Ltd, grants and non-financial support from 10.13039/100004334Merck Sharp & Dohm, grants from 10.13039/501100000771Prostate Cancer UK, grants and non-financial support from Aventis Pharma Limited (Sanofi), outside the submitted work.

AW reports a fellowship from 10.13039/501100000289Cancer Research UK during the conduct of the study as well as subsequent funding from 10.13039/100004325AstraZeneca and imCORE. BG, HT, CG, CS, FD, CC and NS have nothing to disclose.
