# Supplementary material for: Multi-candidate immunohistochemical markers to assess radiation response and prognosis in prostate cancer: results from the CHHiP trial of radiotherapy fractionation
Source: eBioMedicine. 2023 Jan 26;88:104436. doi: 10.1016/j.ebiom.2023.104436 (PMC9900483; doi:10.1016/j.ebiom.2023.104436)
Supplement: Supplementary Tables S1–S15 and Figs. S1 and S2 [file mmc1.docx]

**Supplementary Appendix**

Table of Contents

[Table S1. Antibody panel optimised for use in Trans-CHHiP 2](#_Toc94126856)

[Table S2. Distribution of the matching variables as categorical variables by fractionation schedules in Ki67 data set. 3](#_Toc94126857)

[Table S3. Distribution of the matching variables as categorical variables by fractionation schedules in P53 data set. 3](#_Toc94126858)

[Table S4. Distribution of the matching variables as categorical variables by fractionation schedules in P16 data set. 4](#_Toc94126859)

[Table S5. Distribution of the matching variables as categorical variables by fractionation schedules in PTEN data set. 4](#_Toc94126860)

[Table S6. Distribution of the matching variables as categorical variables by fractionation schedules in Bcl2 data set. 5](#_Toc94126861)

[Table S7. Distribution of the matching variables as categorical variables by fractionation schedules in Geminin data set 5](#_Toc94126862)

[Table S8. Distribution of the matching variables as categorical variables by fractionation schedules in HIF1α data set 6](#_Toc94126863)

[Table S9. Distribution of the matching variables as categorical variables by fractionation schedules in P-CHK1 data set 6](#_Toc94126864)

[Figure S1: Different staining patterns for p53 IHC. A: clonal mutant, B: total mutant, C and D: wild type. A-C 10X, D:20X 7](#_Toc94126865)

[Table S10: Agreement in IHC markers in Part 1 between scoring investigators 1 and 2 for p53, Bcl2, HIF1α, ATM, MRE11 and VEGF. 8](#_Toc94126866)

[Table S11: Agreement in IHC markers in Part 1 between scoring investigators 1 and 2 for PTEN, EGFR, Geminin and p16. 9](#_Toc94126868)

[Figure S2: IHC markers excluded after Part 1. 10](#_Toc94126871)

[Table S12: Distribution of IHC scores between cases and controls and according to fractionation schedule for p53, p16 and Bcl2 11](#_Toc94126872)

[Table S13: Distribution of IHC scores between cases and controls and according to fractionation schedule for HIF1α and pCHK1 12](#_Toc94126873)

[Table S14: Odds ratios for BCR estimated from univariate conditional logistic regression models without and with interaction terms between continuous variables (mean and maximum Ki67, Geminin, HIF1α and p-chk1) and fractionation schedules. 13](#_Toc94126874)

[Table S15: Odds ratios for BCR estimated from univariate conditional logistic regression models without and with interaction terms between categorical variables (p53, p16, PTEN and Bcl-2) and fractionation schedules. 14](#_Toc94126875)

# Table S1. Antibody panel optimised for use in Trans-CHHiP

PTM: DAKO PT module, PC: pressure cooker

# Table S2. Distribution of the matching variables as categorical variables by fractionation schedules in Ki67 data set (PSA: prostate specific antigen).

| **Ki67 data set** | | | | | | | | |
| --- | --- | --- | --- | --- | --- | --- | --- | --- |
|  | **74Gy/37f**  **Total N = 116** | | **60Gy/20f**  **Total N = 98** | | **57Gy/19f**  **Total N = 122** | | **Total**  **Total N = 336** | |
|  | **N** | **%** | **N** | **%** | **N** | **%** | **N** | **%** |
| **PSA (ng/ml)** |  |  |  |  |  |  |  |  |
| <10 | 32 | 27.6 | 38 | 38.8 | 62 | 50.8 | 132 | 39.3 |
| 10- & <20 | 76 | 65.5 | 50 | 51.0 | 50 | 41.0 | 176 | 52.4 |
| 20- | 8 | 6.9 | 10 | 10.2 | 10 | 8.2 | 28 | 8.3 |
| **Tumour stage** |  |  |  |  |  |  |  |  |
| T1 | 22 | 19.0 | 16 | 16.3 | 38 | 31.1 | 76 | 22.6 |
| T2 | 86 | 74.1 | 74 | 75.5 | 74 | 60.7 | 234 | 69.6 |
| T3 | 8 | 6.9 | 8 | 8.2 | 10 | 8.2 | 26 | 7.7 |
| **Gleason Score** |  |  |  |  |  |  |  |  |
| 6 | 12 | 10.3 | 10 | 10.2 | 16 | 13.1 | 38 | 11.3 |
| 3+4 | 62 | 53.4 | 58 | 59.2 | 62 | 50.8 | 182 | 54.2 |
| 4+3 | 32 | 27.6 | 18 | 18.4 | 26 | 21.3 | 76 | 22.6 |
| 8+ | 10 | 8.6 | 12 | 12.2 | 18 | 14.8 | 40 | 11.9 |

# Table S3. Distribution of the matching variables as categorical variables by fractionation schedules in P53 data set (PSA: prostate specific antigen).

| **P53 data set** | | | | | | | | |
| --- | --- | --- | --- | --- | --- | --- | --- | --- |
|  | **74Gy/37f**  **Total N = 114** | | **60Gy/20f**  **Total N = 96** | | **57Gy/19f**  **Total N = 118** | | **Total**  **Total N = 328** | |
|  | **N** | **%** | **N** | **%** | **N** | **%** | **N** | **%** |
| **PSA (ng/ml)** |  |  |  |  |  |  |  |  |
| <10 | 30 | 26.3 | 40 | 41.7 | 56 | 47.5 | 126 | 38.4 |
| 10- & <20 | 76 | 66.7 | 48 | 50 | 52 | 44.1 | 176 | 53.7 |
| 20- | 8 | 7 | 8 | 8.3 | 10 | 8.5 | 26 | 7.9 |
| **Tumour stage** |  |  |  |  |  |  |  |  |
| T1 | 22 | 19.3 | 16 | 16.7 | 34 | 28.8 | 72 | 22 |
| T2 | 84 | 73.7 | 74 | 77.1 | 72 | 61 | 230 | 70.1 |
| T3 | 8 | 7 | 6 | 6.3 | 12 | 10.2 | 26 | 7.9 |
| **Gleason Score** |  |  |  |  |  |  |  |  |
| 6 | 12 | 10.5 | 10 | 10.4 | 16 | 13.6 | 38 | 11.6 |
| 3+4 | 60 | 52.6 | 58 | 60.4 | 64 | 54.2 | 182 | 55.5 |
| 4+3 | 32 | 28.1 | 18 | 18.8 | 22 | 18.6 | 72 | 22 |
| 8+ | 10 | 8.8 | 10 | 10.4 | 16 | 13.6 | 36 | 11 |

# Table S4. Distribution of the matching variables as categorical variables by fractionation schedules in P16 data set (PSA: prostate specific antigen).

| **P16 data set** | | | | | | | | |
| --- | --- | --- | --- | --- | --- | --- | --- | --- |
|  | **74Gy/37f**  **Total N = 94** | | **60Gy/20f**  **Total N = 78** | | **57Gy/19f**  **Total N = 104** | | **Total**  **Total N = 276** | |
|  | **N** | **%** | **N** | **%** | **N** | **%** | **N** | **%** |
| **PSA (ng/ml)** |  |  |  |  |  |  |  |  |
| <10 | 26 | 27.7 | 30 | 38.5 | 46 | 44.2 | 102 | 37 |
| 10- & <20 | 64 | 68.1 | 42 | 53.8 | 50 | 48.1 | 156 | 56.5 |
| 20- | 4 | 4.3 | 6 | 7.7 | 8 | 7.7 | 18 | 6.5 |
| **Tumour stage** |  |  |  |  |  |  |  |  |
| T1 | 16 | 17 | 14 | 17.9 | 32 | 30.8 | 62 | 22.5 |
| T2 | 70 | 74.5 | 60 | 76.9 | 64 | 61.5 | 194 | 70.3 |
| T3 | 8 | 8.5 | 4 | 5.1 | 8 | 7.7 | 20 | 7.2 |
| **Gleason Score** |  |  |  |  |  |  |  |  |
| 6 | 6 | 6.4 | 6 | 7.7 | 18 | 17.3 | 30 | 10.9 |
| 3+4 | 50 | 53.2 | 50 | 64.1 | 52 | 50 | 152 | 55.1 |
| 4+3 | 28 | 29.8 | 14 | 17.9 | 20 | 19.2 | 62 | 22.5 |
| 8+ | 10 | 10.6 | 8 | 10.3 | 14 | 13.5 | 32 | 11.6 |

# Table S5. Distribution of the matching variables as categorical variables by fractionation schedules in PTEN data set (PSA: prostate specific antigen).

| **PTEN data set** | | | | | | | | |
| --- | --- | --- | --- | --- | --- | --- | --- | --- |
|  | **74Gy/37f**  **Total N = 96** | | **60Gy/20f**  **Total N = 92** | | **57Gy/19f**  **Total N = 112** | | **Total**  **Total N =300** | |
|  | **N** | **%** | **N** | **%** | **N** | **%** | **N** | **%** |
| **PSA (ng/ml)** |  |  |  |  |  |  |  |  |
| <10 | 26 | 27.1 | 40 | 43.5 | 54 | 48.2 | 120 | 40 |
| 10- & <20 | 62 | 64.6 | 44 | 47.8 | 48 | 42.9 | 154 | 51.3 |
| 20- | 8 | 8.3 | 8 | 8.7 | 10 | 8.9 | 26 | 8.7 |
| **Tumour stage** |  |  |  |  |  |  |  |  |
| T1 | 14 | 14.6 | 16 | 17.4 | 36 | 32.1 | 66 | 22 |
| T2 | 74 | 77.1 | 72 | 78.3 | 68 | 60.7 | 214 | 71.3 |
| T3 | 8 | 8.3 | 4 | 4.3 | 8 | 7.1 | 20 | 6.7 |
| **Gleason Score** |  |  |  |  |  |  |  |  |
| 6 | 10 | 10.4 | 10 | 10.9 | 16 | 14.3 | 36 | 12 |
| 3+4 | 48 | 50 | 52 | 56.5 | 58 | 51.8 | 158 | 52.7 |
| 4+3 | 28 | 29.2 | 18 | 19.6 | 24 | 21.4 | 70 | 23.3 |
| 8+ | 10 | 10.4 | 12 | 13 | 14 | 12.5 | 36 | 12 |

# Table S6. Distribution of the matching variables as categorical variables by fractionation schedules in Bcl2 data set (PSA: prostate specific antigen).

| **Bcl2 data set** | | | | | | | | |
| --- | --- | --- | --- | --- | --- | --- | --- | --- |
|  | **74Gy/37f**  **Total N = 106** | | **60Gy/20f**  **Total N = 96** | | **57Gy/19f**  **Total N = 108** | | **Total**  **Total N = 310** | |
|  | **N** | **%** | **N** | **%** | **N** | **%** | **N** | **%** |
| **PSA (ng/ml)** |  |  |  |  |  |  |  |  |
| <10 | 28 | 26.4 | 38 | 39.6 | 52 | 48.1 | 118 | 38.1 |
| 10- & <20 | 72 | 67.9 | 50 | 52.1 | 48 | 44.4 | 170 | 54.8 |
| 20- | 6 | 5.7 | 8 | 8.3 | 8 | 7.4 | 22 | 7.1 |
| **Tumour stage** |  |  |  |  |  |  |  |  |
| T1 | 20 | 18.9 | 16 | 16.7 | 34 | 31.5 | 70 | 22.6 |
| T2 | 78 | 73.6 | 72 | 75 | 64 | 59.3 | 214 | 69 |
| T3 | 8 | 7.5 | 8 | 8.3 | 10 | 9.3 | 26 | 8.4 |
| **Gleason Score** |  |  |  |  |  |  |  |  |
| 6 | 12 | 11.3 | 10 | 10.4 | 14 | 13 | 36 | 11.6 |
| 3+4 | 54 | 50.9 | 56 | 58.3 | 56 | 51.9 | 166 | 53.5 |
| 4+3 | 30 | 28.3 | 18 | 18.8 | 22 | 20.4 | 70 | 22.6 |
| 8+ | 10 | 9.4 | 12 | 12.5 | 16 | 14.8 | 38 | 12.3 |

# Table S7. Distribution of the matching variables as categorical variables by fractionation schedules in Geminin data set (PSA: prostate specific antigen).

| **Geminin data set** | | | | | | | | |
| --- | --- | --- | --- | --- | --- | --- | --- | --- |
|  | **74Gy/37f**  **Total N = 104** | | **60Gy/20f**  **Total N = 94** | | **57Gy/19f**  **Total N = 110** | | **Total**  **Total N = 308** | |
|  | **N** | **%** | **N** | **%** | **N** | **%** | **N** | **%** |
| **PSA (ng/ml)** |  |  |  |  |  |  |  |  |
| <10 | 28 | 26.9 | 36 | 38.3 | 54 | 49.1 | 118 | 38.3 |
| 10- & <20 | 70 | 67.3 | 48 | 51.1 | 46 | 41.8 | 164 | 53.2 |
| 20- | 6 | 5.8 | 10 | 10.6 | 10 | 9.1 | 26 | 8.4 |
| **Tumour Stage** |  |  |  |  |  |  |  |  |
| T1 | 18 | 17.3 | 14 | 14.9 | 36 | 32.7 | 68 | 22.1 |
| T2 | 78 | 75 | 74 | 78.7 | 64 | 58.2 | 216 | 70.1 |
| T3 | 8 | 7.7 | 6 | 6.4 | 10 | 9.1 | 24 | 7.8 |
| **Gleason Score** |  |  |  |  |  |  |  |  |
| 6 | 10 | 9.6 | 10 | 10.6 | 16 | 14.5 | 36 | 11.7 |
| 3+4 | 54 | 51.9 | 56 | 59.6 | 58 | 52.7 | 168 | 54.5 |
| 4+3 | 30 | 28.8 | 16 | 17 | 22 | 20 | 68 | 22.1 |
| 8+ | 10 | 9.6 | 12 | 12.8 | 14 | 12.7 | 36 | 11.7 |

# Table S8. Distribution of the matching variables as categorical variables by fractionation schedules in HIF1α data set (PSA: prostate specific antigen).

| **HIF1α data set** | | | | | | | | |
| --- | --- | --- | --- | --- | --- | --- | --- | --- |
|  | **74Gy/37f**  **Total N = 110** | | **60Gy/20f**  **Total N = 92** | | **57Gy/19**  **Total N = 108** | | **Total**  **Total N = 310** | |
|  | **N** | **%** | **N** | **%** | **N** | **%** | **N** | **%** |
| **PSA (ng/ml)** |  |  |  |  |  |  |  |  |
| <10 | 30 | 27.3 | 40 | 43.5 | 52 | 48.1 | 122 | 39.4 |
| 10- & <20 | 74 | 67.3 | 44 | 47.8 | 48 | 44.4 | 166 | 53.5 |
| 20- | 6 | 5.5 | 8 | 8.7 | 8 | 7.4 | 22 | 7.1 |
| **Tumour Stage** |  |  |  |  |  |  |  |  |
| T1 | 24 | 21.8 | 16 | 17.4 | 32 | 29.6 | 72 | 23.2 |
| T2 | 78 | 70.9 | 70 | 76.1 | 66 | 61.1 | 214 | 69 |
| T3 | 8 | 7.3 | 6 | 6.5 | 10 | 9.3 | 24 | 7.7 |
| **Gleason Score** |  |  |  |  |  |  |  |  |
| 6 | 12 | 10.9 | 8 | 8.7 | 12 | 11.1 | 32 | 10.3 |
| 3+4 | 58 | 52.7 | 56 | 60.9 | 60 | 55.6 | 174 | 56.1 |
| 4+3 | 32 | 29.1 | 16 | 17.4 | 22 | 20.4 | 70 | 22.6 |
| 8+ | 8 | 7.3 | 12 | 13 | 14 | 13 | 34 | 11 |

# Table S9. Distribution of the matching variables as categorical variables by fractionation schedules in P-CHK1 data set (PSA: prostate specific antigen).

| **P-CHK1 data set** | | | | | | | | |
| --- | --- | --- | --- | --- | --- | --- | --- | --- |
|  | **74Gy/37f**  **Total N = 102** | | **60Gy/20f**  **Total N = 86** | | **57Gy/19**  **Total N = 110** | | **Total**  **Total N = 298** | |
|  | **N** | **%** | **N** | **%** | **N** | **%** | **N** | **%** |
| **PSA (ng/ml)** |  |  |  |  |  |  |  |  |
| <10 | 28 | 27.5 | 38 | 44.2 | 54 | 49.1 | 120 | 40.3 |
| 10- & <20 | 68 | 66.7 | 40 | 46.5 | 48 | 43.6 | 156 | 52.3 |
| 20- | 6 | 5.9 | 8 | 9.3 | 8 | 7.3 | 22 | 7.4 |
| **Tumour Stage** |  |  |  |  |  |  |  |  |
| T1 | 18 | 17.6 | 16 | 18.6 | 38 | 34.5 | 72 | 24.2 |
| T2 | 76 | 74.5 | 64 | 74.4 | 64 | 58.2 | 204 | 68.5 |
| T3 | 8 | 7.8 | 6 | 7 | 8 | 7.3 | 22 | 7.4 |
| **Gleason Score** |  |  |  |  |  |  |  |  |
| 6 | 10 | 9.8 | 8 | 9.3 | 16 | 14.5 | 34 | 11.4 |
| 3+4 | 54 | 52.9 | 50 | 58.1 | 58 | 52.7 | 162 | 54.4 |
| 4+3 | 28 | 27.5 | 18 | 20.9 | 22 | 20 | 68 | 22.8 |
| 8+ | 10 | 9.8 | 10 | 11.6 | 14 | 12.7 | 34 | 11.4 |

# Figure S1: Different staining patterns for p53 IHC. A: clonal mutant, B: total mutant, C and D: wild type. A-C 10X, D:20X


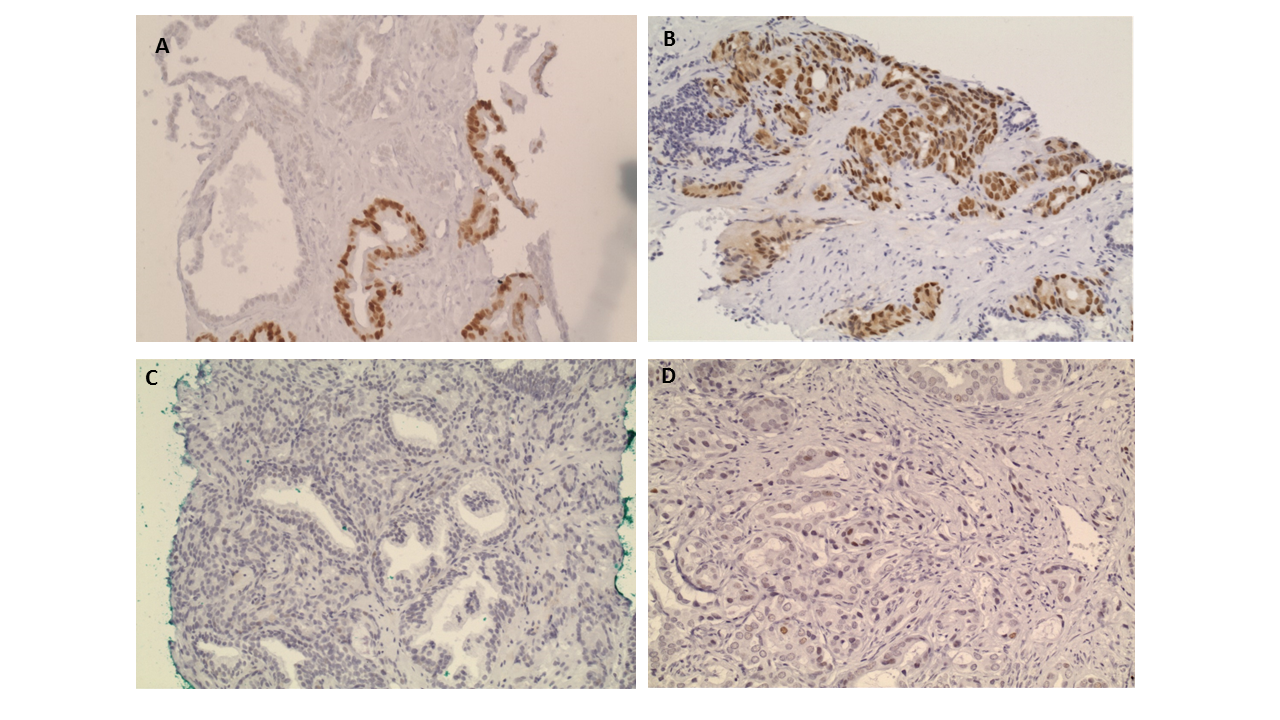


# Table S10: Agreement in IHC markers in Part 1 between scoring investigators 1 and 2 for p53, Bcl2, HIF1α, ATM, MRE11 and VEGF.

# *less than 110 slides were stained due to lack of tissue

# Table S11: Agreement in IHC markers in Part 1 between scoring investigators 1 and 2 for PTEN, EGFR, Geminin and p16.

## *less than 110 slides were stained due to lack of tissue

## ^$^Mixed score for p16 included at least 100 cells ≤25% and at least 100 cells >25%

**
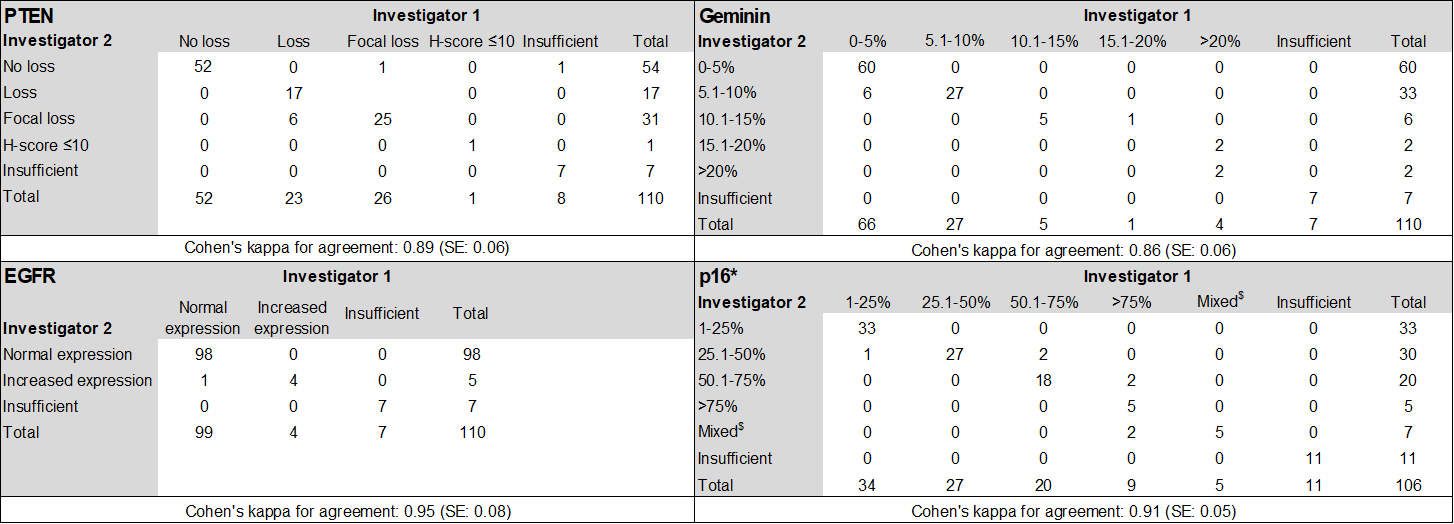
**

Figure S2: IHC markers excluded after Part 1. **Homogenous staining in normal prostate and tumour is shown for ATM in nuclei (A) and VEGF in cytoplasm (B). Heterogeneous cytoplasmic staining within cases is shown for OPN (C) and COX2 (D). A:10X, B-D:20X**


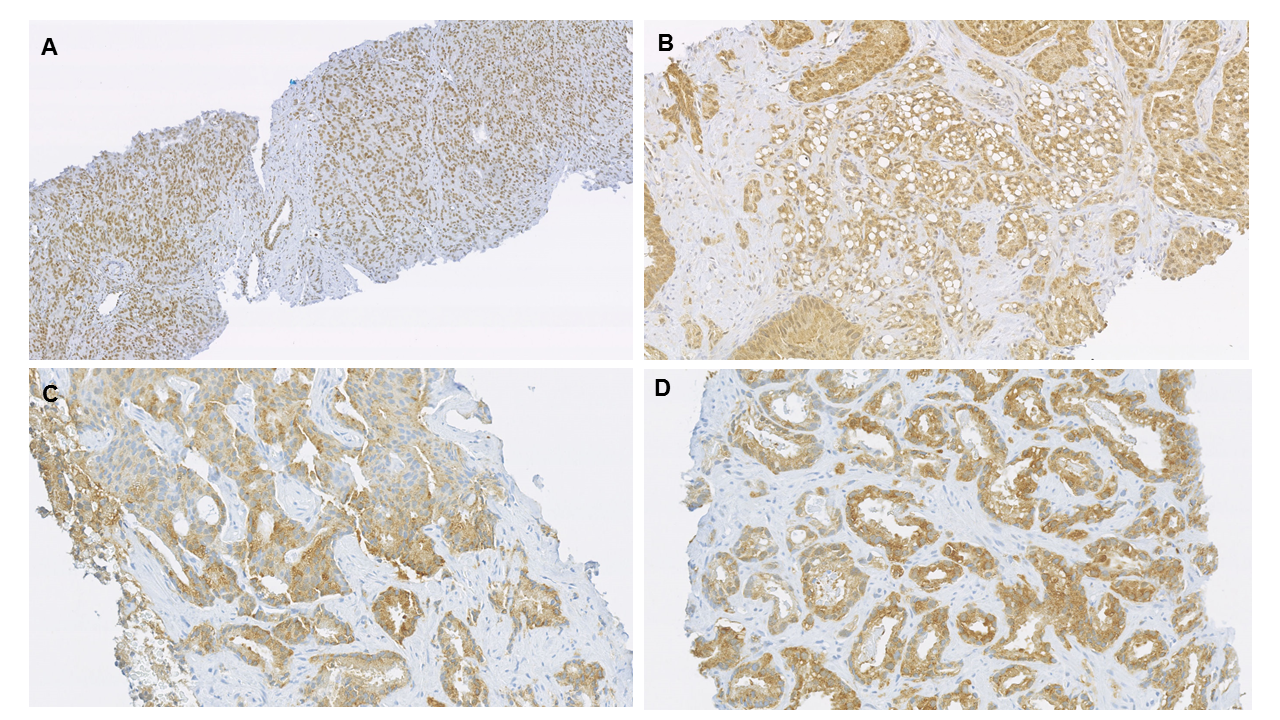


# Table S12: Distribution of IHC scores between cases and controls and according to fractionation schedule for p53, p16 and Bcl2

# Table S13: Distribution of IHC scores between cases and controls and according to fractionation schedule for HIF1α and pCHK1

## **Table S14: Odds ratios for BCR estimated from univariate conditional logistic regression models without and with interaction terms between continuous variables (mean and maximum Ki67, Geminin, HIF1α and p-chk1) and fractionation schedules**.

| **Schedules** | **OR** | **95 % CI (OR)** | **P value (OR)** | **P value for interaction*** |
| --- | --- | --- | --- | --- |
| **mean Ki67** |  |  |  |  |
| 74 Gy & 60 Gy | 1.09 | 1.02 – 1.17 | 0.007 | 0.26 |
| 74 Gy & 57 Gy | 1.07 | 1.01 – 1.14 | 0.03 | 0.59 |
| 60 Gy & 57 Gy | 1.11 | 1.04 – 1.19 | 0.001 | 0.59 |
| **max Ki67** |  |  |  |  |
| 74 Gy & 60 Gy | 1.06 | 1.02 – 1.11 | 0.008 | 0.34 |
| 74 Gy & 57 Gy | 1.04 | 1.00 – 1.08 | 0.08 | 0.82 |
| 60 Gy & 57 Gy | 1.06 | 1.01 – 1.11 | 0.02 | 0.27 |
| **Geminin** |  |  |  |  |
| 74 Gy & 60 Gy | 1.14 | 1.05 - 1.25 | 0.003 | 0.56 |
| 74 Gy & 57 Gy | 1.05 | 0.98 - 1.13 | 0.2 | 0.06 |
| 60 Gy & 57 Gy | 1.04 | 0.98 - 1.12 | 0.21 | 0.1 |
| **ln(HIF1α)** |  |  |  |  |
| 74 Gy & 60 Gy | 0.57 | 0.13 - 2.46 | 0.45 | n/a |
| 74 Gy & 57 Gy | 1.13 | 0.56 - 2.3 | 0.74 | 0.27 |
| 60 Gy & 57 Gy | 1.6 | 0.54 - 4.75 | 0.4 | n/a |
| **P-chk1** |  |  |  |  |
| 74 Gy & 60 Gy | 1.24 | 0.8 - 1.91 | 0.34 | 0.27 |
| 74 Gy & 57 Gy | 1.2 | 0.72 - 1.98 | 0.48 | 0.11 |
| 60 Gy & 57 Gy | 0.82 | 0.48 - 1.4 | 0.47 | 0.5 |

* Odds ratios (OR) are adjusted for the matching variables and age at randomisation. As no values are significant, p-values for the BH adjustment with 10% False Discovery Rate are not included.

# Table S15: Odds ratios for BCR estimated from univariate conditional logistic regression models without and with interaction terms between categorical variables (p53, p16, PTEN and Bcl-2) and fractionation schedules.

| **Schedules** |  | **OR** | **95 % CI (OR)** | **P value (OR)** | **P value for interaction*** |
| --- | --- | --- | --- | --- | --- |
| **p53** |  |  |  |  |  |
| 74 Gy & 60 Gy | Wild type | 1 |  |  |  |
|  | Mutant | 1 | 0.32 - 3.11 | 1 | 0.56 |
| 74 Gy & 57 Gy | Wild type | 1 |  |  |  |
|  | Mutant | 1.4 | 0.44 - 4.42 | 0.57 | 0.92 |
| 60 Gy & 57 Gy | Wild type | 1 |  |  |  |
|  | Mutant | 1.04 | 0.3 - 3.61 | 0.95 | 0.47 |
| **p16** |  |  |  |  |  |
| 74 Gy & 60 Gy | Wild type | 1 |  |  |  |
|  | Mutant | 0.61 | 0.34 - 1.1 | 0.1 | 0.89 |
| 74 Gy & 57 Gy | Wild type | 1 |  |  |  |
|  | Mutant | 0.71 | 0.42 - 1.23 | 0.22 | 0.67 |
| 60 Gy & 57 Gy | Wild type | 1 |  |  |  |
|  | Mutant | 0.72 | 0.4 - 1.3 | 0.28 | 0.67 |
| **PTEN** |  |  |  |  |  |
| 74 Gy & 60 Gy | Wild type | 1 |  |  |  |
|  | Mutant | 2.19 | 1.21 - 3.97 | 0.01 | 0.26 |
| 74 Gy & 57 Gy | Wild type | 1 |  |  |  |
|  | Mutant | 2.74 | 1.53 - 4.9 | 0.0007 | 0.69 |
| 60 Gy & 57 Gy | Wild type | 1 |  |  |  |
|  | Mutant | 1.94 | 1.09 - 3.43 | 0.02 | 0.42 |
| **Bcl-22** |  |  |  |  |  |
| 74 Gy & 60 Gy | No increase | 1 |  |  |  |
|  | Increase | 0.85 | 0.39 - 1.87 | 0.69 | 0.4 |
| 74 Gy & 57 Gy | No increase | 1 |  |  |  |
|  | Increase | 0.68 | 0.28 - 1.65 | 0.39 | 0.74 |
| 60 Gy & 57 Gy | No increase | 1 |  |  |  |
|  | Increase | 1.03 | 0.45 - 2.36 | 0.95 | 0.71 |

* Odds ratios (OR) are adjusted for the matching variables and age at randomisation
